# Change points, memory and epidemic spreading in temporal networks

**DOI:** 10.1038/s41598-018-33313-1

**Published:** 2018-10-19

**Authors:** Tiago P. Peixoto, Laetitia Gauvin

**Affiliations:** 1 Department of Mathematical Sciences and Centre for Networks and Collective Behaviour, University of Bath, Claverton Down, Bath, BA2 7AY United Kingdom; 2 ISI Foundation, Via Chisola 5, 10126 Torino, Italy

## Abstract

Dynamic networks exhibit temporal patterns that vary across different time scales, all of which can potentially affect processes that take place on the network. However, most data-driven approaches used to model time-varying networks attempt to capture only a single characteristic time scale in isolation — typically associated with the short-time memory of a Markov chain or with long-time abrupt changes caused by external or systemic events. Here we propose a unified approach to model both aspects simultaneously, detecting short and long-time behaviors of temporal networks. We do so by developing an arbitrary-order mixed Markov model with change points, and using a nonparametric Bayesian formulation that allows the Markov order and the position of change points to be determined from data without overfitting. In addition, we evaluate the quality of the multiscale model in its capacity to reproduce the spreading of epidemics on the temporal network, and we show that describing multiple time scales simultaneously has a synergistic effect, where statistically significant features are uncovered that otherwise would remain hidden by treating each time scale independently.

## Introduction

Recent advances in the study of network systems — usually with social, technological and biological origins — have been moving beyond the more traditional approach of considering them as static or growing entities, and instead have been introducing more realistic descriptions that allow them to change arbitrarily in time^1,2^. This effort includes modeling of the time-varying network structure^3,4^, as well as processes that take place on this dynamic environment, such as epidemic spreading^5–8^. Further recent works^9–11^ have highlighted the role of memory, burstiness and time ordering as key features of empirical temporal networks that affect dynamical processes taking place on it.

Most approaches, however, rely on a characteristic time scale on which they describe the dynamics. These can be divided, roughly, into approaches that model temporal correlations via Markov chains relating short-time memory with future behavior^12,13^, and those that model the dynamics at longer times, usually via network snapshots^14–19^ or discrete change points^20–22^. For example, in Refs.^12,13^ the time evolution of a network is represented as a static Markov chain where the placement of new edges is conditioned on the last few edges placed. Since the transition probabilities themselves do not change in time, the system eventually reaches equilibrium and cannot maintain any kind of long-term memory. Conversely, the approaches of Refs.^14–22^ do not attempt to model any kind of short term memory, and simply divide the temporal evolution into discrete intervals, according to how large is the change in the network structure between these intervals. In so doing, these approaches focus only on a larger temporal scale, describing only abrupt changes in the large-scale network structure. In reality, however, most systems exhibit both kinds of dynamics, and focusing on a single aspect comes at the expense of ignoring the other. In this work, we introduce a data-driven modeling approach that includes both aspects simultaneously, and is capable of uncovering both the short-time Markov properties as well a the long-time abrupt changes.

We develop a Bayesian formulation that allows both the change points and the Markov order to be inferred from data in a principled manner, prevents overfitting and enables model selection. As an extraneous evaluation of our approach, we investigate the behavior of epidemic spreading both in the original data and in artificial ones generated from our inferred models. We show that the most plausible models tend to mix both short-time memory and many change points, and those tend to capture well the nontrivial epidemic behavior observed in the original data. Importantly, the inferred models with change points typically uncover higher-order memory than the simpler stationary variants, demonstrating that the mixed approach is more powerful than considering individual ones in isolation.

This paper is divided as follows. In Sec. 2.1 we present the epidemic models that will be used for the model comparison. In Sec. 2.2 we describe our modeling and inferring approach, and apply it to empirical data. In Sec. 4 we finalize with a conclusion.

## Results

### Proximity networks and epidemic dynamics

In the interest of simplicity, we will consider a minimal model of temporal networks and epidemic dynamics that takes place on it. The most central simplification we will make is that the dynamics takes place in discrete time, so that the placement of edges forms a temporal sequence, where only one edge is placed at any given time. Real dynamical networks and epidemic spreading occur in continuous time, but our objective here is not to construct a detailed realistic model, but rather to illustrate how multiple time scales can be described simultaneously. More realistic features can then be added to the model at a later stage.

More specifically, we consider temporal networks composed of *N* nodes, where the placement of the edges occurs sequentially in time, i.e. they define a sequence ***s*** = {*x*_*t*_}, where *x*_*t*_ = (*u*, *v*)_*t*_ is an edge between nodes *u* and *v* observed at time *t*, with *t* = {1, 2, …, *E*}, where *E* is the total number of edge occurrences, and the number of nodes *N* remains constant. Although this formulation is general, we focus in particular on proximity networks, obtained by tracking volunteers with wearable sensors over a period of time^23–26^, so that an edge (*u*, *v*)_*t*_ is recorded if the respective people came closer than a given radius at time *t*. Data recorded in this manner possess enough time resolution for our analysis, and also serve as a plausible scenario for epidemic spreading^27^.

In the above scenario, we assume that an infection can only occur at time *t* over the current “active” edge (*u*, *v*)_*t*_. If the epidemics follows the Susceptible-Infected-Recovered (SIR) model, and *σ*_*u*_(*t*) ∈ {S, I, R} is the state of node *u* at time *t*, we have at each time step *t*:

1. If (*u*, *v*)_*t*_ is the current edge, with (*σ*_*u*_(*t* − 1), *σ*_*v*_(*t* − 1)) = (*S*, *I*) or (*I*, *S*), the infection spreads with probability *β*, so that (*σ*_*u*_(*t*), *σ*_*v*_(*t*)) = (*I*, *I*).
2. For every infected node *u* with *σ*_*u*_(*t* − 1) = *I*, it becomes recovered *σ*_*u*_(*t*) = *R* with probability *γ*.

The parameters *β* and *γ* control the infection and recovery probabilities, respectively. We also consider the Susceptible-Infected-Susceptible (SIS) model, which is a variation of the above, where in the second step the infected nodes become susceptible, *σ*_*u*_(*t*) = *S*, instead of recovered. In both cases, we consider the total number of infected nodes at given time *t*, *X*(*t*). For any positive recovery probability *γ* > 0, the long-time behavior of the SIR model is always ${\mathrm{lim}}_{t\to \infty }X(t)=0$, as the outbreak invariably dies out, whereas in the SIS model it can persist for arbitrarily long times in large systems. In the following, we will use the behavior of *X*(*t*) as a proxy for the comparison between data and model in capturing the underlying network dynamics.

When considering epidemics on dynamical networks, there are two properties that are believed to be crucial for the spreading process^10,11^: 1. The distribution of number of contacts per link, i.e. the frequency of token *x* in sequence ***s***, and 2. The distribution of waiting (or inter-event) times, i.e. the time between two occurrences of the same edge. Although a link that occurs frequently is likely to have shorter inter-event times, the latter tends to vary in ranges that cannot be explained fully by the former, and represents temporal correlations that go beyond the mere frequency of occurrence of edges^10,11^. We will have these two aspects in mind when elaborating our models.

### Models for temporal networks

Our objective is to construct a generative model for temporal networks that includes both short-term memories and abrupt change points. We begin by formulating a stationary version, without change points, and show how it is insufficient to capture many features in the data. We then extend the model to include change points, and perform a comparison.

#### Stationary Markov chains

We consider sequences of discrete tokens, i.e. edges, ***s*** = {*x*_*t*_} with *t* ∈ {1, …, *E*} being by definition both the time and the number of edges that have been placed, and *x*_*t*_ ∈ {1, …, *D*} the set of unique edges with cardinality *D*, which are generated from a stationary Markov chain of order *n*, i.e. they occur with probability

1 $$P({\boldsymbol{s}}|{\boldsymbol{p}},n)=\prod _{t}{p}_{{x}_{t},{{\boldsymbol{x}}}_{t-1}}=\prod _{x,{\boldsymbol{x}}}{p}_{x,{\boldsymbol{x}}}^{{a}_{x,{\boldsymbol{x}}}},$$

where ***p*** corresponds to the transition matrix and ${p}_{{x}_{t},{{\boldsymbol{x}}}_{t-1}}$ is the probability of observing token *x*_*t*_ given the previous *n* tokens ***x***_*t*−1_ = {*x*_*t*−1_, …, *x*_*t*−*n*_} in the sequence, and *a*_*x*,***x***_ is the number of observed transitions from memory ***x*** to token *x*. This serves a simple model for temporal networks, where each possible token corresponds to an edge in the network, i.e. *x*_*t*_ ≡ (*i*, *j*)_*t*_, as we considered previously. Despite its simplicity, this model is able to reproduce arbitrary edge frequencies, determined by the steady-state distribution of the tokens *x*, as well as causal temporal correlations between edges. This means that the model should be able to reproduce properties of the data that can be attributed to the distribution of number of contacts per link, which are believed to be important for epidemic spreading^10,11^. However, due to its Markovian nature, the dynamics will eventually forget past states, and converge to the limiting distribution (assuming the chain is ergodic and aperiodic). This latter property means that the model should be able to capture nontrivial statistics of waiting times only at a short time scale, comparable to the Markov order.

Given the above model, the simplest way to proceed would be to infer transition probabilities from data using maximum likelihood, i.e. maximizing Eq. 1 under the normalization constraint ${\sum }_{x}\,{p}_{x,{\boldsymbol{x}}}=1$. This yields

2 $${\hat{p}}_{x,{\boldsymbol{x}}}=\frac{{a}_{x,{\boldsymbol{x}}}}{{k}_{{\boldsymbol{x}}}}.$$

where ${k}_{{\boldsymbol{x}}}={\sum }_{x}\,{a}_{x,{\boldsymbol{x}}}$ is the number of transitions originating from ***x***. However, if we want to determine the most appropriate Markov order *n* that fits the data, the maximum likelihood approach cannot be used, as it will *overfit*, i.e. the likelihood of Eq. 1 will increase monotonically with *n*, favoring the most complicated model possible, and thus confounding statistical fluctuations with actual structure. Instead, the most appropriate way to proceed is to consider the Bayesian posterior distribution

3 $$P(n|{\boldsymbol{s}})=\frac{P({\boldsymbol{s}}|n)P(n)}{P({\boldsymbol{s}})},$$

which involves the integrated marginal likelihood^28^

4 $$P({\boldsymbol{s}}|n)=\int P({\boldsymbol{s}}|{\boldsymbol{p}},n)P({\boldsymbol{p}}|n)\,{\rm{d}}{\boldsymbol{p}},$$

where the prior probability *P*(***p***|*n*) encodes the amount of knowledge we have on the transitions ***p*** before we observe the data. If we possess no information, we can be agnostic by choosing a uniform prior

5 $$P({\boldsymbol{p}}|n)=\prod _{{\boldsymbol{x}}}(D-\mathrm{1)!}\delta (1-\sum _{x}\,{p}_{x,{\boldsymbol{x}}}),$$

which assumes that all transition probabilities are equally likely. Inserting Eqs. 1 and 5 in Eq. 4, and calculating the integral we obtain

6 $$P({\boldsymbol{s}}|n)=\prod _{{\boldsymbol{x}}}\,\frac{(D-\mathrm{1)!}}{({k}_{{\boldsymbol{x}}}+D-\mathrm{1)!}}\prod _{x}\,{a}_{x,{\boldsymbol{x}}}\mathrm{!.}$$

The remaining prior, *P*(*n*), that represents our *a priori* preference to the Markov order, can also be chosen in an agnostic fashion in a range [0, *N*], i.e.

7 $$P(n)=\frac{1}{N+1}.$$

Since this prior is a constant, the upper bound *N* has no effect on the posterior of Eq. 3, provided it is sufficiently large to include most of the distribution.

Differently from the maximum-likelihood approach described previously, the posterior distribution of Eq. 3 will select the size of the model to match the statistical significance available, and will favor a more complicated model only if the data cannot be suitably explained by a simpler one, i.e. it corresponds to an implementation of Occam’s razor that prevents overfitting.

**Figure 1:**
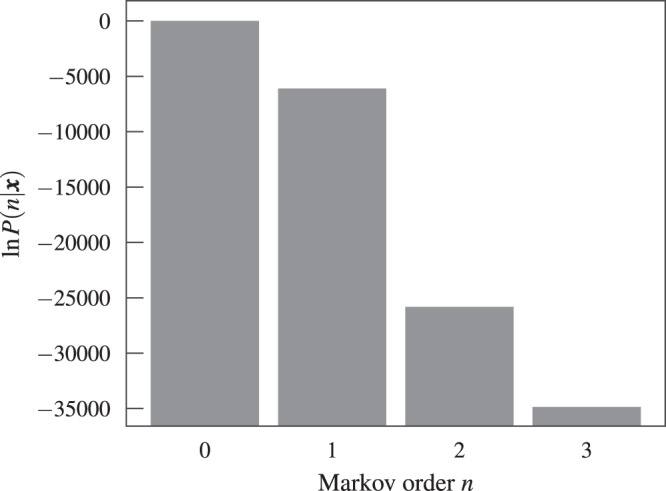
Posterior distribution of the Markov order *P*(*n*|***x***) (Eq. 3) for a temporal network between students in a high school^36^.

**Figure 2:**
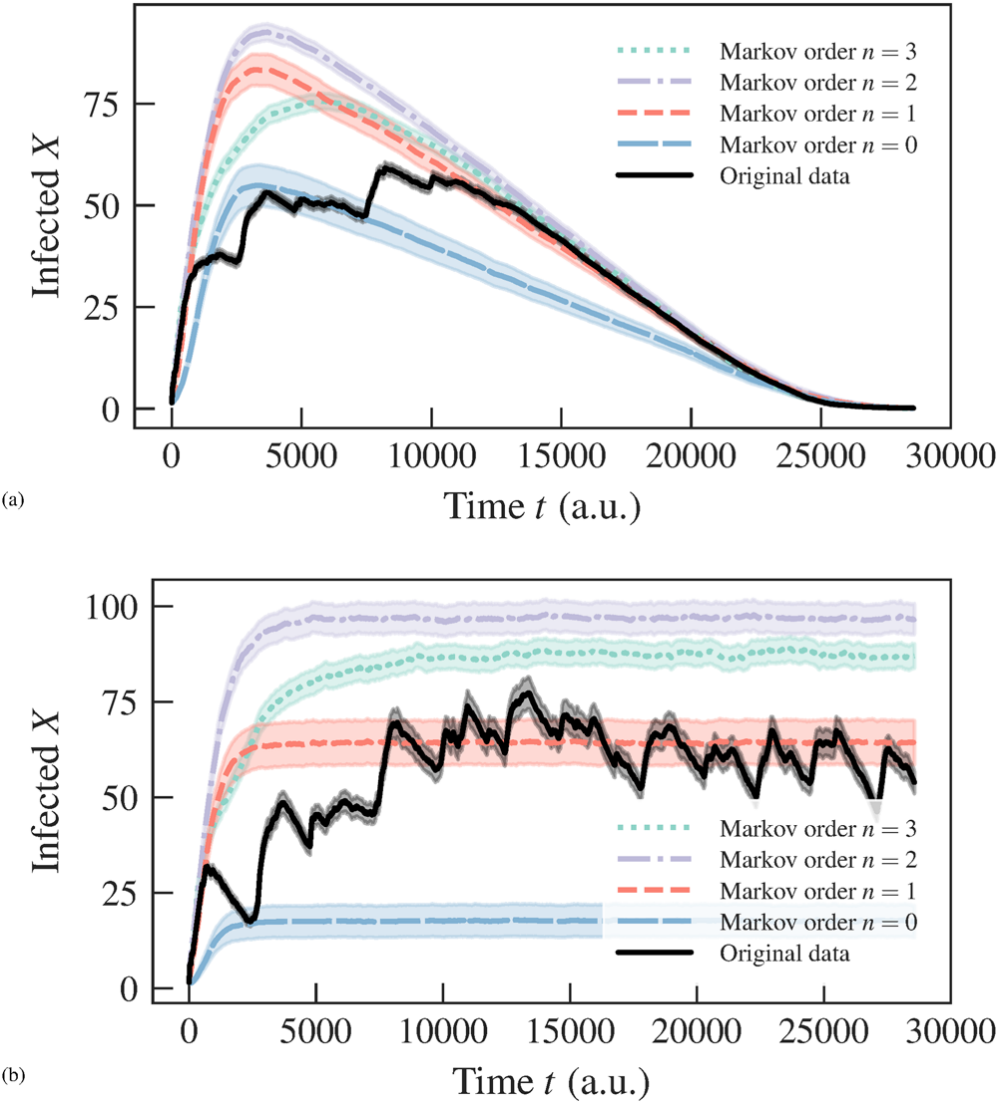
Number of infected nodes over time *X*(*t*) for a temporal network between students in a high-school^36^ (*N* = 126), considering both the original data and artificial time-series generated from the fitted Markov model of a given order *n*, using (**a**) SIR (*β* = 0.41, *γ* = 0.005) and (**b**) SIS (*β* = 0.61, *γ* = 0.03) epidemic models. In all cases, the values were averaged over 100 independent realizations of the network model (for the artificial datasets) and dynamics. The shaded areas indicate the standard deviation of the mean. The values of the infection and recovery rates were chosen so that the spreading dynamics spans the entire time range of the dataset.

When applying this approach to empirical data, we observe that it favors *n* = 0 for all datasets we considered (not shown), indicating that a higher-order model is not statistically justified, as can be seen in Fig. 1. However, if we generate temporal networks from the fitted models, i.e. sequence of edges using the transition probabilities ${\hat{p}}_{x,{\boldsymbol{x}}}$ = *a*_*x*,***x***_/*k*_***x***_, they exhibit epidemic dynamics that are very different from what we observe on the empirical time-series, as can be seen in Fig. 2: for the original data, the epidemic spreading is marked by abrupt changes in the infection rate, which are not reproduced by the model for any value of Markov order *n* — even those that overfit. Therefore, these patterns in the epidemic dynamics seem to stem from changes in the underlying structure of the temporal network that are not captured by the above Markov model. Among other things, this means that the behavior cannot be explained by a heterogeneous distribution of edge frequencies, as this is well described by the model. As we show in the next section, the situation changes considerably once we generalize the model to incorporate heterogeneous Markov chains with change points.

#### Markov chains with change points

We attempt to model the abrupt changes observed in the previous section by non-stationary transition probabilities *p*_*x*,***x***_ that change abruptly at a given “change point,” but otherwise remain constant between change points. The occurrence of change points is governed by the probability *q* that one is inserted at any given time. The existence of *M* change points divide the time series into *M* + 1 temporal segments indexed by *l* ∈ {0, …, *M*}. The variable *l*_*t*_ indicates to which temporal segment a given time *t* belongs among the *M* segments. Thus, the conditional probability of observing a token *x* at time *t* in segment *l*_*t*_ is given by

8 $$P({x}_{t},{l}_{t}|{{\boldsymbol{x}}}_{t-1},{l}_{t-1})={p}_{x,{\boldsymbol{x}}}^{{l}_{t}}[q(1-{\delta }_{{l}_{t},{l}_{t-1}})+(1-q){\delta }_{{l}_{t},{l}_{t-1}}],$$

where ${p}_{x,{\boldsymbol{x}}}^{{l}_{t}}$ is the transition probability inside segment *l*_*t*_ and *q* is the probability to transit from segment *l* to *l* + 1. The probability of a whole sequence ***s*** = {*x*_*t*_} and ***l*** = {*l*_*t*_} being generated is then

9 $$P({\boldsymbol{s}},{\boldsymbol{l}}|{\boldsymbol{p}},q)={q}^{M}{\mathrm{(1}-q)}^{E-M}\prod _{l,x,{\boldsymbol{x}}}\,{({p}_{x,{\boldsymbol{x}}}^{l})}^{{a}_{x,{\boldsymbol{x}}}^{l}}$$

where ${a}_{x,{\boldsymbol{x}}}^{l}$ is the number of transitions from memory ***x*** to token *x* in the segment *l*. Note that we recover the stationary model of Eq. 1 by setting *q* = 0. The maximum-likelihood estimates of the parameters are

10 $${\hat{p}}_{x,{\boldsymbol{x}}}^{l}=\frac{{a}_{x,{\boldsymbol{x}}}^{l}}{{k}_{{\boldsymbol{x}}}^{l}},\,\hat{q}=\frac{M}{E}$$

where ${k}_{{\boldsymbol{x}}}^{l}={\sum }_{x}\,{a}_{x,{\boldsymbol{x}}}^{l}$ is the number of transitions originating from ***x*** in a segment *l*. But once more, we want to infer the model the segments ***l*** in a Bayesian way, via the posterior distribution

11 $$P({\boldsymbol{l}}|{\boldsymbol{s}},n)=\frac{P({\boldsymbol{s}},{\boldsymbol{l}}|n)}{P({\boldsymbol{s}}|n)},$$

where the numerator is the integrated likelihood

12 $$P({\boldsymbol{s}},{\boldsymbol{l}}|n)=\int P({\boldsymbol{s}},{\boldsymbol{l}}|{\boldsymbol{p}},q,n)P({\boldsymbol{p}}|n)P(q)\,{\rm{d}}\,{\boldsymbol{p}}\,\,{\rm{d}}q$$

using uniform priors *P*(*q*) = 1, and

13 $$P({\boldsymbol{p}}|n)=\prod _{l}P({{\boldsymbol{p}}}_{l}|{d}_{l},n)P({d}_{l}),$$

with the uniform prior

14 $$P({{\boldsymbol{p}}}_{l}|{d}_{l},n)=\prod _{x}({D}_{l}-1)!\delta (1-\sum _{x}{p}_{x,x}^{l}).$$

and

15 $$P({d}_{l})={2}^{-D}$$

being the prior for the alphabet *d*_*l*_ of size *D*_*l*_ inside segment *l*, sampled uniformly from all possible subsets of the overall alphabet of size *D*. Performing the above integral, we obtain

16 $$P({\boldsymbol{x}},{\boldsymbol{l}}|n)={2}^{-D(M+\mathrm{1)}}\frac{M!(E-M)!}{(E+\mathrm{1)}!}\prod _{l}\prod _{{\boldsymbol{x}}}\frac{({D}_{l}-\mathrm{1)}!}{({k}_{{\boldsymbol{x}}}^{l}+{D}_{l}-\mathrm{1)}!}\prod _{x}{a}_{x,{\boldsymbol{x}}}^{l}!.$$

Like with the previous stationary model, both the order and the positions of the change points can be inferred fr**o**m the joint posterior distribution

17 $$P({\boldsymbol{l}},n|{\boldsymbol{x}})=\frac{P({\boldsymbol{x}},{\boldsymbol{l}}|n)P(n)}{P({\boldsymbol{x}})},$$

in a manner that intrinsically prevents overfitting. This constitutes a robust and elegant way of extracting this information from data, that contrasts with non-Bayesian methods of detecting change points using Markov chains that tend to be more cumbersome^29^, and is more versatile than approaches that have a fixed Markov order^30^.

The exact computation of the posterior of Eq. 11 would require the marginalization of the above distribution for all possible segments ***l***, yielding the denominator *P*(***x***|*n*), which is unfeasible for all but the smallest time series. However, it is not necessary to compute this value if we sample ***l*** from the posterior using Monte Carlo. We do so by making move proposals ***l*** → ***l***′ with probability *P*(***l***′|***l***), and accepting it with probability *a* according to the Metropolis-Hastings criterion^31,32^

18 $$a=\,\min (1,\frac{P({\boldsymbol{l}}^{\prime} |{\boldsymbol{x}},n)P({\boldsymbol{l}}|{\boldsymbol{l}}^{\prime} )}{P({\boldsymbol{l}}|{\boldsymbol{x}},n)P({\boldsymbol{l}}^{\prime} |{\boldsymbol{l}})}),$$

which does not require the computation of *P*(***x***|*n*) as it cancels out in the ratio. If the move proposals are ergodic, i.e. they allow every possible partition ***l*** to be visited eventually, this algorithm will asymptotically sample from the desired posterior. Here we use the following move proposal scheme, choosing between one the following actions with equal probability:

1. We select a segment randomly and split it in a random point in the middle.
2. We merge two adjacent segments.
3. We move a randomly chosen boundary to a random position between the two enclosing ones.

We perform this algorithm many times, starting from a single segment, and waiting sufficiently long for equilibration — determined by observing if the likelihood value no longer changes significantly — and we choose the partition with the largest probability across runs. For all datasets we investigated, we observed a fast convergence of this algorithm, which typically shows very little variation between runs.

Note that it is also possible to change the Markov order during the algorithm, by proposing moves *n* → *n*′, and using the Metropolis-Hastings criterion to accept or reject them. However, we found that Markov order typically settles very early in the algorithm, and no longer changes during the remaining run, as it incurs a macroscopic change in the likelihood. Since changing the Markov order is an expensive operation of order *O*(*E*), we have found it is best to leave it fixed during the MCMC, and select it later according to the likelihood value.

**Figure 3:**
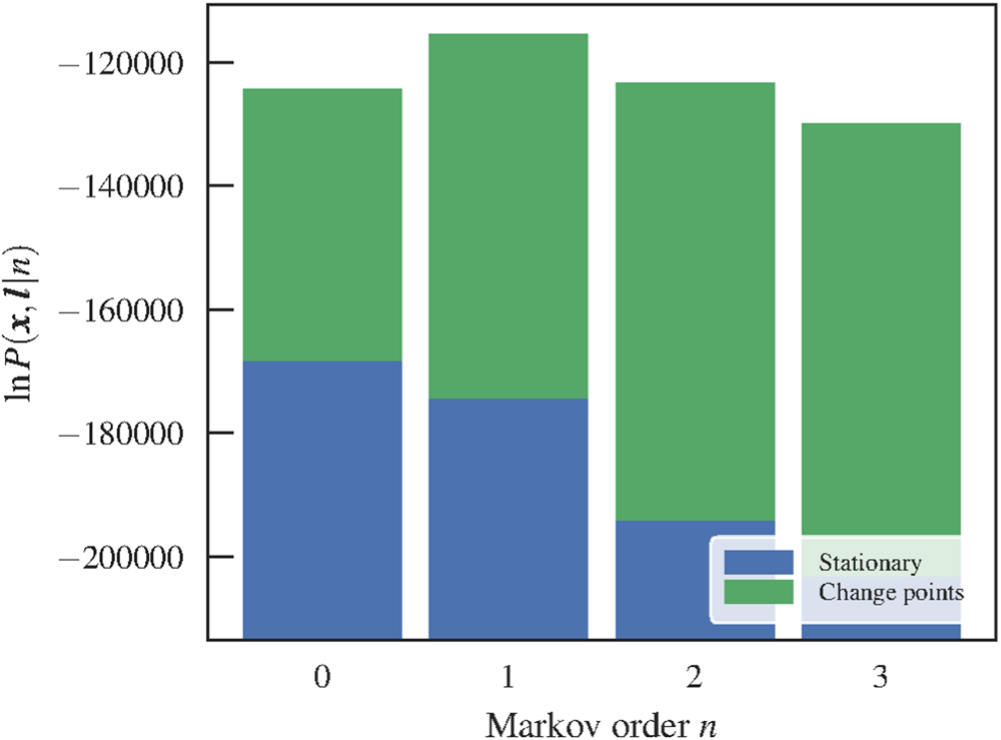
Integrated joint likelihood *P*(***x***, ***l***|*n*) (Eq. 16) for a temporal network between students in a high school^36^, for the stationary (i.e. zero change points) and nonstationary models. For all values of *n* the likelihoods are higher for the nonstationary model (yielding a posterior odds ratio Λ > 1).

Once a fit is obtained, we can compare the above model with the stationary one by computing the posterior odds ratio

19 $${\rm{\Lambda }}=\frac{P({\boldsymbol{l}},n|{\boldsymbol{x}})}{P({{\boldsymbol{l}}}_{0},{n}_{0}|{\boldsymbol{x}})}=\frac{P({\boldsymbol{x}},{\boldsymbol{l}}|n)}{P({\boldsymbol{x}},{{\boldsymbol{l}}}_{0}|{n}_{0})},$$

where ***l***_0_ is the partition into a single interval (which is equivalent to the stationary model). A value Λ > 1 [i.e. *P*(***x***, ***l***|*n*) > *P*(***x***, ***l***_0_|*n*_0_)] indicates a larger evidence for the nonstationary model. As can be seen in Fig. 3, we observe indeed a larger evidence for the nonstationary model for all Markov orders. In addition to this, using this general model we identify *n* = 1 as the most plausible Markov order, in contrast to the *n* = 0 obtained with the stationary model. Therefore, identifying change points allows us not only to uncover patterns at longer time scales, but the separation into temporal segments enables the identification of statistically significant patterns at short time scales as well, which would otherwise remain obscured with the stationary model — even though it is designed to capture only these kinds of correlations.

**Figure 4:**
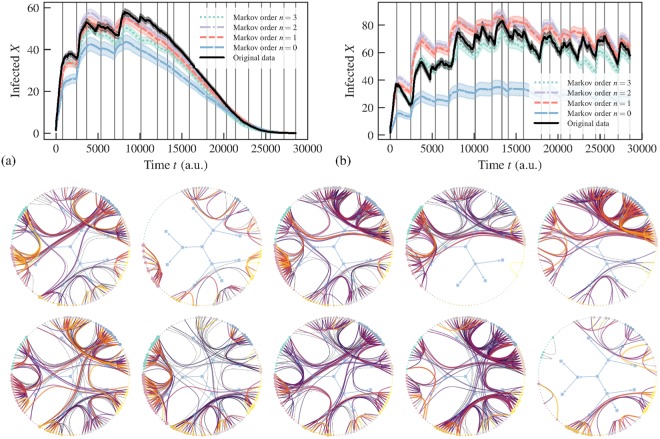
(Above) Number of infected nodes over time *X*(*t*) for a temporal network between students in a high-school^36^ (*N* = 126), considering both the original data and artificial time-series generated from the fitted nonstationary Markov model of a given order *n*, using (**a**) SIR (*β* = 0.41, *γ* = 0.005) and (**b**) SIS (*β* = 0.61, *γ* = 0.03) epidemic models. The vertical lines mark the position of the inferred change points. In all cases, the values were averaged over 100 independent realizations of the network model (for the artificial datasets) and dynamics. The shaded areas indicate the standard deviation of the mean. (Below) Network structure inside the first ten segments, as captured by a layered hierarchical degree-corrected stochastic block model^16^ using the frequency of interactions as edge covariates^33^ (indicated by colors), where each segment is considered as a different layer. The values of the infection and recovery rates were chosen so that the spreading dynamics spans the entire time range of the dataset.

The improved quality of this model is also evident when we investigate the epidemic dynamics, as shown in Fig. 4. In order to obtain an estimate of the number of infected based on the model, we generated different sequences of edges using the fitted segments and transition probabilities ${\hat{p}}_{x,{\boldsymbol{x}}}^{l}={a}_{x,{\boldsymbol{x}}}^{l}/{k}_{{\boldsymbol{x}}}^{l}$ in each of the segments estimated with Markov orders going from 0 to 3. We simulated SIR and SIS processes on top of the networks generated and averaged the number of infected over many instances. Looking at Fig. 4, we see that the inferred positions of the change-points tend to coincide with the abrupt changes in infection rates, which show very good agreement between the empirical and generated time-series. For higher Markov order, the agreement improves, although the improvement seen for *n* > 1 is probably due to overfitting, given the results of Fig. 3. We note also that the fact that *n* = 0 provides the worse fit and agreement with epidemic dynamics shows that it is not only the existence of change points, but also the inferred Markov dynamics that contribute to the quality of the model in reproducing the epidemic spreading.

In order to examine the link between the structure of the network and the change points, we fitted a layered hierarchical degree-corrected stochastic block model^16,33^ to the data, considering each segment as a separate edge layer. From the figure Fig. 4) we can see that the density of connections between node groups vary in a substantial manner, suggesting that change point marks an abrupt transition in the typical kind of encounters between students — representing breaks between classes, meal time, etc (see Fig. 4). This yields an insight as to why these changes in pattern may slow down or speed up an epidemic spreading: if students are confined to their classrooms, contagion across classrooms is inhibited, but as soon they are free to move around the school grounds, so can the epidemic.

**Figure 5:**
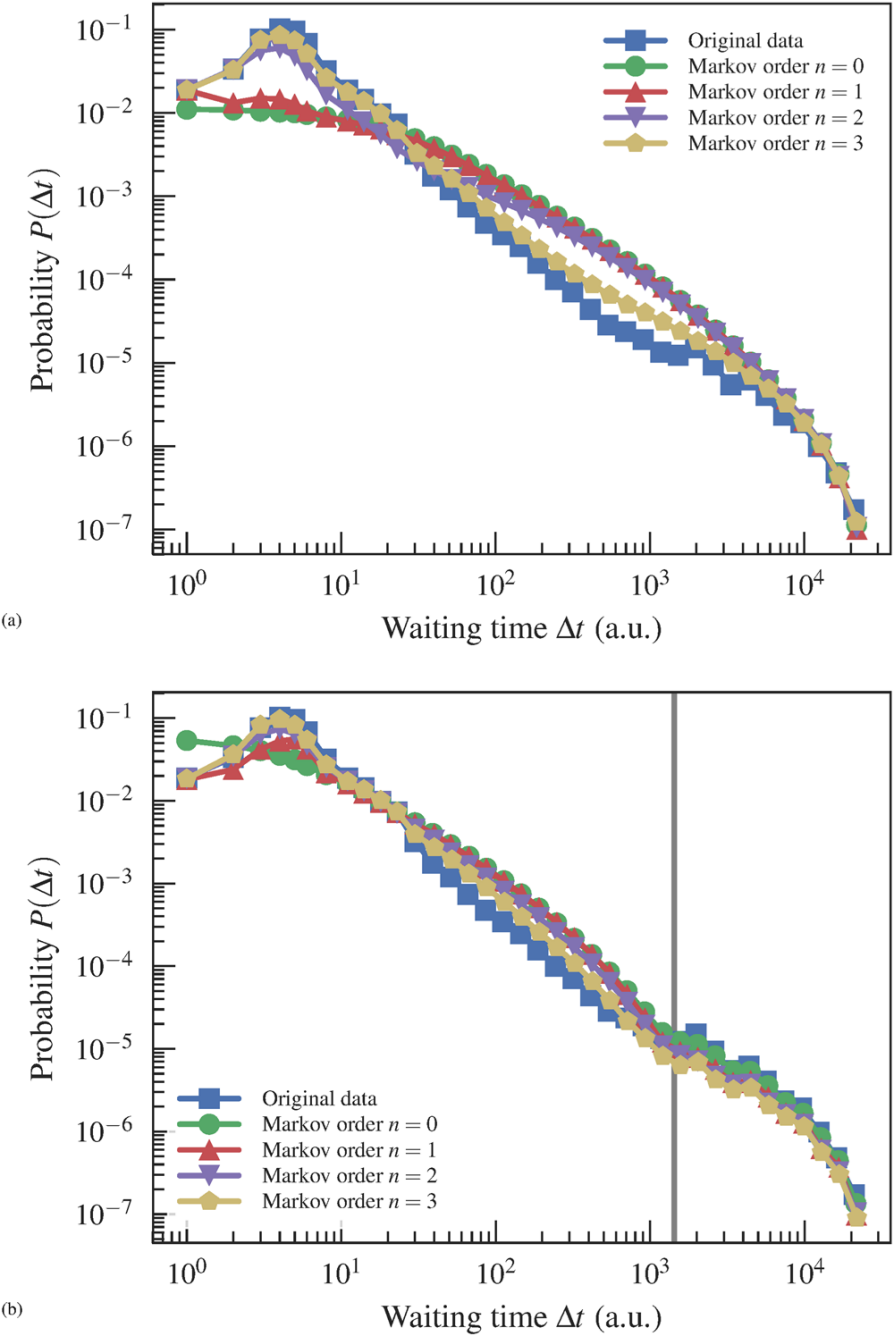
Distribution of waiting times Δ*t* between the same edge for the empirical dataset and fitted (**a**) stationary and (**b**) nonstationary models (a single instance of each), for a temporal network between students in a high school^36^. The vertical line shows the average length of inferred stationary segments between change points.

We explore further the match between data and model by measuring the distribution of waiting times between temporal edges, i.e. the time interval between the occurrence in the time series of the same edge in the network, shown in Fig. 5 for both Markov models. For the empirical dataset, the waiting time distribution shows a characteristic peak at short times, and a broad decay for longer ones. For the stationary model, the distributions obtained with the fitted models show significant discrepancy — for both long and short times — except when the Markov order is increased to *n* = 3, which, according to our Bayesian analysis cannot be used as an explanation for the data, as it represents an overfit. However, for the nonstationary model with change points, we observe a fair agreement between data and model for the most-likely model with *n* = 1, across all time scales. The nonstationary model also provides an explanation to the shape of the distribution at longer times, which shows a separation of time scales inside individual stationary segments, from larger ones across change points (marked as vertical line in Fig. 5). In addition to this, the fact that the *n* = 0 model does not reproduce the short time behavior of the distribution shows that the Markov property inside each stationary segment is indeed a necessary ingredient of the model. The model that best fits the data is able to reproduce with a quite good degree of approximation the distribution of waiting times, across all time scales. This point is in agreement with previous results highlighting the importance of the heterogeneity of inter-event times for dynamical processes^34^, but here we see how two different time scales are sufficient to reproduce a large fraction of the observed behavior.

In Sec. 3 we show that the same behavior is obtained for a variety of different datasets.

## Other datasets

**Figure 6:**
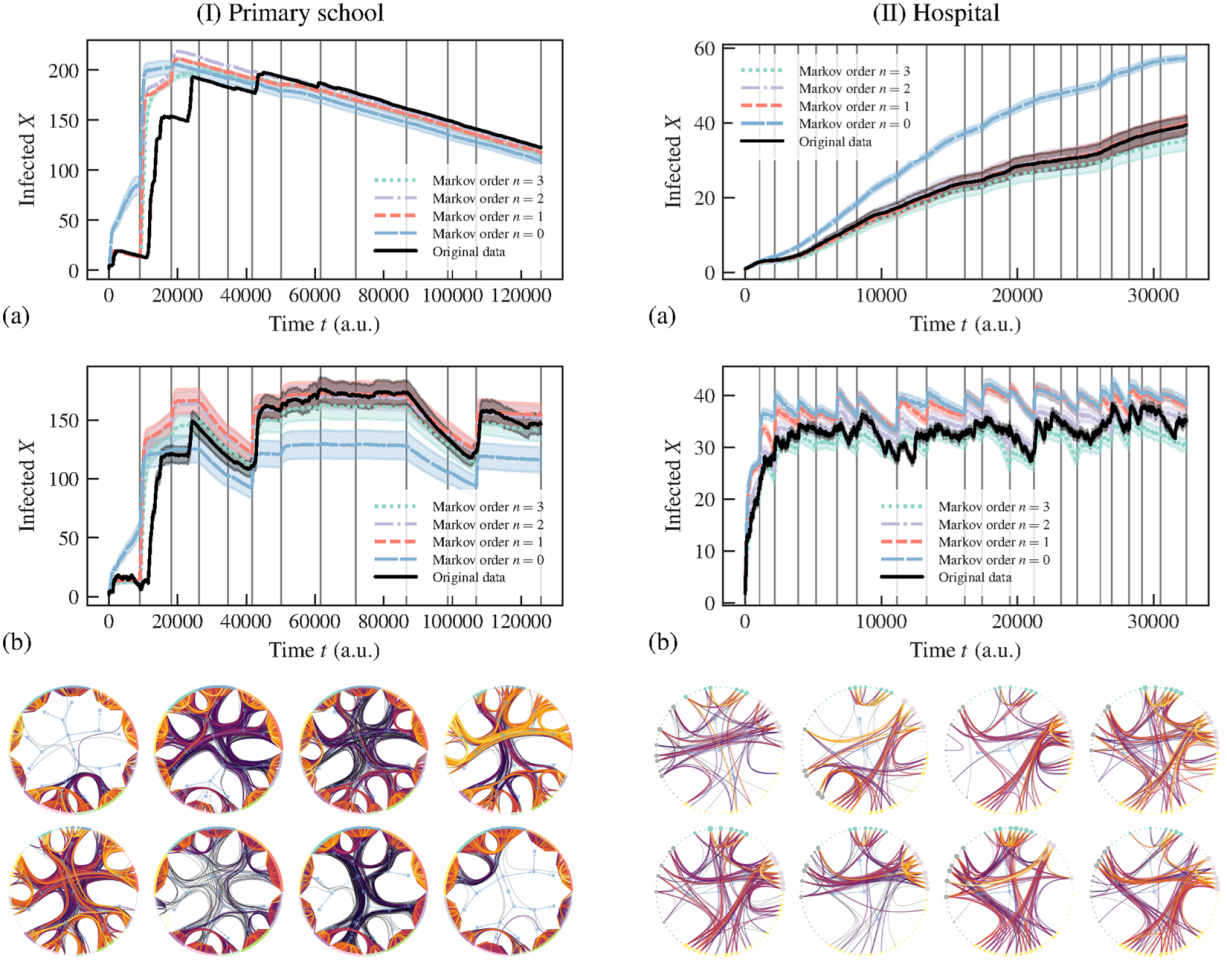
(Above) Number of infected nodes over time *X*(*t*) for temporal networks between (I) students in a primary school^24^ (*N* = 242) and (II) patients and staff of a hospital^25^ (*N* = 75), considering both the original data and artificial time-series generated from the fitted nonstationary Markov model of a given order *n*, using (**a**) SIR [(I) *β* = 0.9, *γ* = 0.001; (II) *β* = 0.001, *γ* = 0] and (**b**) SIS [(I) *β* = 0.84, *γ* = 0.01; (II) *β* = 0.81, *γ* = 0.015] epidemic models. The vertical lines mark the position of the inferred change points. In all cases, the values were averaged over 100 independent realizations of the network model (for the artificial datasets) and dynamics. The shaded areas indicate the standard deviation of the mean. (Below) Network structure inside the first eight segments, as captured by a layered hierarchical degree-corrected stochastic block model^16^ using the frequency of interactions as edge covariates^33^ (indicated by colors), where each segment is considered as a different layer. The values of the infection and recovery rates were chosen so that the spreading dynamics spans the entire time range of the dataset.

Here we show that very similar results to those described above are also encountered for other proximity datasets. In Fig. 6(I) we show the analysis for the temporal behavior of students in a primary school^24^, which shows a very clear correlation of the change in infection rate and the inferred change points. If we inspect the network structure inside each temporal segment, we see that amounts to periods of time where the students are either confined into classes, or mingling in larger groups. A similar behavior is seen if Fig. 6(II) for people (staff and patients) in a hospital ward^25^.

## Discussion

In this work we presented a data-driven approach to model temporal networks that is based on the simultaneous description of the network dynamics in two time scales: 1. The occurrence of the edges according to an arbitrary-order Markov chain, 2. The abrupt transition of the Markov transition probabilities at specific change-points. We developed a Bayesian framework that allows the inference of the change points and Markov order from data in manner that prevents overfitting, and enables the selection of competing models.

We have applied our approach to a variety of empirical proximity networks, and we have evaluated the inferred models based on their capacity to reproduce the epidemic spreading observed with the original data. We have seen that the nonstationary model accurately reproduces the highly-variable nature of the infection rate, with changes correlating strongly with the inferred change points. Furthermore, we showed that the inferred model also accurately reproduces the waiting time statistics in the empirical data, both at small and large time scales, neither of which are accurately captured if the different time scales are analyzed in isolation.

We argue that, ultimately, the incorporation of such temporal heterogeneity is indispensable for the evaluation of the speeding up or slowing down of processes taking place on dynamic networks^12,35^, and the development of mitigating strategies against epidemics^27^.

Although our model successfully captures key properties of real dynamic networks, it can still be made more realistic in a variety of ways. For instance, it can be extended to continuous time via the incorporation of waiting time distributions between events, as done in ref.^13^. Furthermore, it remains also to be seen how the approach presented here can be extended to scenarios where edges are allowed both to appear and disappear from the network, so that its dynamics can no longer be represented simply by a sequence of edges. And lastly, it would be desirable to provide a more direct connection between the edge probabilities and change points with large-scale network descriptors, such as community structure.

## Data Availability

The datasets generated during analysed during the current study are available in the sociopatterns website, at http://www.sociopatterns.org.
